# Salubridad y enfermedad en las minas de oro de Marmato en el siglo XIX: entre la epidemia de viruela y la guerra de los mil días

**DOI:** 10.7705/biomedica.5945

**Published:** 2021-10-15

**Authors:** Fernando Gómez, Sebastián López, Arnoby Chacón

**Affiliations:** 1 Departamento Clínico, Facultad de Ciencias para la Salud, Universidad de Caldas, Manizales, Colombia Universidad de Caldas Departamento Clínico Facultad de Ciencias para la Salud Universidad de Caldas Manizales Colombia; 2 Programa de Investigaciones en Gerontología y Geriatría, Universidad de Caldas, Manizales, Colombia Universidad de Caldas Programa de Investigaciones en Gerontología y Geriatría Universidad de Caldas Manizales Colombia

**Keywords:** salubridad ambiental, miner**í**a, historia, enfermedades profesionales, medicina tropical, Environmental healthiness, mining, history, occupational diseases, tropical medicine

## Abstract

La minería ha tenido una gran influencia en las sociedades humanas, permeando por igual las riquezas del suelo y la cultura, lo que ha tenido profundas implicaciones para los individuos dedicados a esta labor y para los lugares en los que se lleva a cabo. En el presente artículo, se describen las características socioculturales y de sanidad, así como las enfermedades más frecuentes en las minas de oro de Marmato (Caldas) durante el siglo XIX. Las precarias condiciones de salubridad y las enfermedades tropicales infecciosas persistieron en la población durante todo el siglo.

## Abstract (en)

Mining has had a great influence on human societies permeating the riches of the soil and culture in equal proportion. This has led to profound changes in the individuals dedicated to this work and the locations where it takes place.

In this historical review, we describe the socio-cultural and health characteristics, as well as the diseases associated with gold mining in Marmato (Caldas) during the 19th century. Poor salubrity conditions and tropical and infectious diseases were constant during the whole century.

A comienzos del siglo XVI, los españoles descubrieron las laderas avolcanadas donde hoy está situado el municipio de Marmato, entre Caldas y Antioquia (figura 1). En los primeros siglos, se conoció como Moroga o Moraga, y fue célebre por la gran cantidad de oro en sus filones ^1^. Desde el siglo XVI fue asiento de minas subterráneas de oro elemental, explotadas de manera rudimentaria por pequeños grupos de españoles y mestizos, con pequeñas cuadrillas de esclavos negros huidos de Popayán ^2^. En 1787, las tres principales regiones auríferas de la Nueva Granada eran Antioquia, Popayán y Chocó. Antes de la fundación de Riosucio en 1819, San Juan de Marmato, la Vega de Supía y el Real de Minas de Quiebralomo, eran el eje de la riqueza minera de esa zona ^3^. En 1824, las minas de Marmato se arrendaron a compañias inglesas como garantía de los empréstitos del gobierno inglés, las cuales mantuvieron el control sobre ellas hasta comienzos del siglo XX ^4^. En 1872, pobladores provenientes de “los departamentos del Sur de Antioquia” como parte de la colonización antioqueña en el occidente de Caldas, refundaron la antigua ciudad colonial de Anserma (Santa Ana de los Caballeros), se diseminaron por la región y, junto con los negros, mestizos, mulatos y los escasos indios sobrevivientes, fueron protagonistas de la vida regional ^5^. Esta zona pertenecía al Estado Soberano del Cauca, pero siempre se consideró parte de los “departamentos de Antioquía” y estuvo ligada a su historia, como lo refiere en su memoria sobre la medicina en los departamentos antioqueños el doctor Emilio Robledo, quien situaba su frontera sur “hasta donde moja sus robustos tarsos en el río de la Vieja (Cartago) de linfas mansas” ^6^. Las disputas por tierras y minas en el cantón de Supía eran el reflejo de la confrontación política y económica que vivía Colombia en siglo XIX, época en la cual las guerras civiles y escaramuzas fueron constantes ^7^.

**Figura 1:**
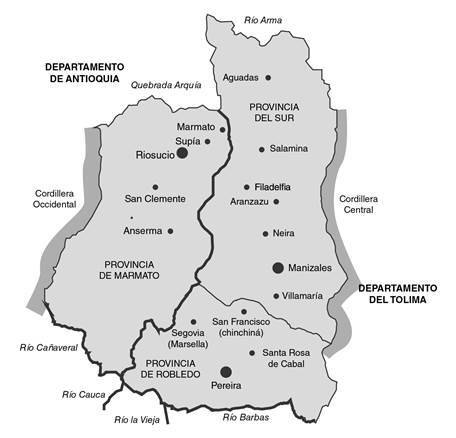
Departamento de Caldas, comienzos del siglo XX

Fuente: https://godues.wordpress.com/2012/11/13/mapas-del-departamento-de-caldas-en-colombia/

## Aspectos de salubridad

A finales del siglo XVIII, los pobladores indígenas ya no eran los mismos grupos del siglo XVI (armados, paucuras, pozos, picaras, carrapas y quimbayas, cartamas, caramantas, ansermas e irrás), sino la mezcla y reagrupamiento de los sobrevivientes y los desplazados de estas tribus, a quienes la minería y la expropiación de las tierras de labranza llevó a padecer hambre.

Sobre la población de Marmato en los años siguientes hay gran controversia, pues algunos autores afirman que para 1850 se contaba con 7.000 pobladores en las minas, incluidos los extranjeros que laboraban allí ^8^. Jean-Baptiste Boussingault -el primer administrador de las minas- describe que en 1830 los habitantes eran cerca de 3.000 y, *“*todos libres, vivían en el declive de la montaña” ^9^. Sin embargo, en su *Geografía médica y nosológica del departamento de Caldas*, Robledo afirma que la población era mucho menor, de alrededor de 1.057 en 1843 y de 2.811 en 1870 ^1^.

Los relatos sobre las condiciones socioambientales en el Marmato del siglo XIX describen algunos factores determinantes en salud: dificultades climáticas, pésimas vías de comunicación, malas condiciones de vivienda de los esclavos, hacinamiento, poca disponibilidad y deficiente calidad de los víveres, inadecuadas condiciones en el interior de las minas, problemas de salud perinatal, falta de aseo e higiene personal, además de violencia intrafamiliar ^2^.

La referencia a los determinantes de salud y a las enfermedades de esta población proviene de lo señalado por el inspector real y oidor Juan Antonio Mon y Velarde Cienfuegos y Valladares (llamado padre y “Regenerador de Antioquia”), quien visitó la zona en 1788 y publicó un documento que permite comprender las características sociales y sanitarias de la región ^10^.

Para ejemplificar las características malsanas del clima, durante el traslado de “la santa Imagen de la Reyna de los Angeles”, enviada como regalo por Felipe II, el cura Joaquín González de Santiago de Arma de Rionegro (1786) decía que el clima era tan malsano en la región, que,

“[…] apenas se puede conservar la Hostia, de un día a otro … siendo de admirar que en tantos años como lleva de cura Esteban Leonín de Estrada, (hombre decrépito y maníaco) en el sitio de Arma Viejo, no huviese observado la particularidad, de humedecerse y arrugarse la santa forma […]” ^10^.

Con respecto a la infraestructura vial, el coronel John Potter Hamilton, agente confidencial de su majestad británica, quien viajó por el país entre 1824 y 1825, menciona que el obstáculo más serio para cualquier actividad explotadora en la región eran “Los pésimos, diré mejor horrorosos caminos de la región, pues entre Cartago y Antioquia (probablemente Arma) se gastan 6 días por la orilla de Cauca” ^11^.

También, era evidente el problema de la vivienda:

“[…] la mala distribución de las casas y la confusión de la familia, viviendo hijos, padres y extraños sin división alguna, es el funesto principio de anticiparse muchas veces la malicia, cometiéndose execrables torpezas […]”,

refiriéndose a embarazos, enfermedades de transmisión sexual y al hacinamiento, que aumentaba el riesgo de enfermedades infectocontagiosas como la tuberculosis (común entre la población minera de la región) ^10^. De hecho, las normas policiales indicaban que en cada casa vivían dos o tres familias sin piezas o divisiones, “todos confundidos y desnudos” y pedía que vivieran separados, o con cuartos con división de sexos, edades y estados “todos como corresponde” ^10^. En los Reales de Minas existían las rancherías, conjunto de ranchos donde vivían los mineros sin separación de sexo o de familia, con otros espacios dedicados a la cocina, las herramientas y la herrería, otro para guardar la sal y los alimentos, y otro con un cepo para el castigo de los esclavos desobedientes ^12^. Desde la segunda década del siglo XIX, la ranchería de Marmato tuvo un rancho para atención de mineros enfermos, y el capataz y los lugartenientes vivían en construcciones separadas. A finales del siglo XVIII,

“[…] Marmato tenía alrededor de 3 o 4 mil habitantes, viviendo en casuchas muy pobres, dispersas en la cresta del farallón, sin orden, sin calles, sin agua corriente, y sin autoridades. No había escuela primaria ni capilla, y probablemente debido al abandono existente, tampoco había autoridades […]” ^2^.

Ocasionalmente aparecía un sacerdote, procedente de la capilla de San Juan de Marmato, a confesar y repartir la comunión, así como comerciantes de hierro, víveres y lienzos. No obstante, los que tenían mayor contacto con los mineros eran los indígenas, lo cual era fundamental para su supervivencia, pues los abastecían especialmente de maíz.

Otro problema era la consecución de suministros y víveres, pues eran traídos desde Cartago, Mariquita y Antioquia e incluían “sal nitro” (nitrato de sodio) para preservar carnes, sal, panela, aguardiente, cerdos y ganado vacuno ^5^. En épocas de buen tiempo, llegar a Marmato desde Cartago tomaba una semana, lo que dificultaba el abastecimiento y el mantenimiento nutricional de la población, como lo describe Mon y Velarde:

“[...] y los infelices mineros y demás pobres que se dedican a este penible ejercicio, o tienen que abandonar la mayor parte del año para hacer rozas y coger su fruto, o tienen que conducir los víveres más precisos a tan larga distancia y por caminos tan ásperos, que importa más su acarreto que el costo principal […]” ^10^.

El Oidor insistía que esto se debía a lo “mal racionado de las cuadrillas”, el hambre, “el corto producto de las minas”, la pobreza que llevaba a “la ruina de los mineros, el atraso del comercio y el abandono de la agricultura” ^10^, queja esta que fue constante durante todo el siglo XIX.

**Figura 2:**
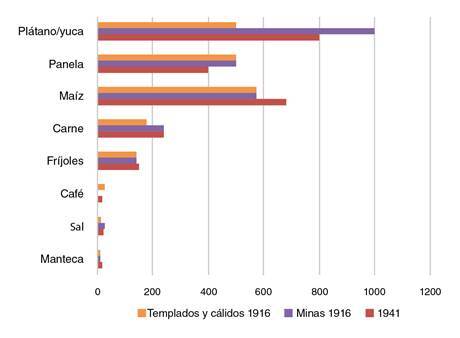
Comparación entre las raciones alimentarias de los mineros y las personas de climas templados y cálidos en 1916 y 1941

Dadas las dificultades para la obtención y movilización de víveres, se esperaría que el contenido nutricional fuera deficiente, sin embargo, la relación entre las raciones suministradas en climas templados y cálidos en 1916 y las de 1941 (figura 2), evidencia un alto consumo de proteínas, indicador del tipo de trabajo realizado y de una mejor calidad de vida ^13^. La yuca se consumía más en las zonas mineras, por ser más resistente a la sequía y por su mayor rendimiento, lo cual era una ventaja para su distribución quincenal en las minas ^5^. Generalmente, los ancianos y los niños esclavos de las rancherías cultivaban yuca y plátano, y las esclavas ancianas cocinaban y asistian a los enfermos ^12^.

La condición de los esclavos no era diferente a la que la literatura refiere en el siglo XVIII, pues no recibían salarios, la alimentación era la que “Dios proveyera”, y la salud y la seguridad eran impensables. Así lo describía el Oidor:

“[…] los continuos recursos de quexas que he tenido, promovidos por varios esclavos, ya por el inhumano y bárbaro tratamiento que reciben de sus amos… y ya por la escases de la ración con que se les contribuye, tolerándoles por esta causa el trabajo en días festivos, con punible abandono del pasto espiritual […]”,

y proponía a los Cabildos varias soluciones ^10^: establecer horas y sitios de trabajo; determinar la ración de comida, diferenciando entre casados y solteros; definir el tipo de vestuario, la periodicidad de cambio y su calidad, y consolidar los castigos que debían imponerse, con lo que debía aumentar la productividad y disminuirse las “alteraciones sociales” asociadas:

“[…] Lo poco útil que será un esclavo mal comido, peor dormido y desnudo, se disminuye su fuerza, incluyendo su capacidad reproductiva con menor descendencia y con predisposición a robar y a asesinar a su amo, o a escaparse […]” ^10^.

Frente a las condiciones perinatales, el Oidor informa sobre: “los muchos infanticidios y repetidos abortos que se experimentan por querer muchas por este punible medio ocultar su fragilidad a los ojos del mundo” ^14^. Era común encontrar madres solteras en las zonas mineras y una esclava podía ser la madre de niños de diferentes padres, incluidos indígenas, blancos y esclavos; sin embargo, era más frecuente el nacimiento de zambos, por cruce entre negros esclavos e indígenas. La mujer era el eje del funcionamiento de las rancherías y las labores diarias. Un siglo después, Ferdinand von Schenck habla de la gran cantidad de negras que trabajaban en las minas y que se amarraban los cortos vestidos a manera de pantalones; a pesar de su tosco físico, eran preferidas por los empleadores de las minas porque superaban en rendimiento a los hombres en los duros trabajos de la minería ^15^.

La violencia intrafamiliar era frecuente; en Santa Bárbara (Antioquia), por ejemplo, en 1788 se “había notado la relajación de costumbres en que se vive, así por el mal trato que dan los maridos a las mujeres, de que han sido muchas y lastimosas las quejas que se han oído…” ^10^. La violencia era constante en las zonas mineras y siempre se señaló que

“[…] estas regiones atraen a muchas gentes sin ocupación ni destino, vagantes y muy nocivas a la sociedad pública, como dispuestas a todo género de vicios, fomentando juegos, riñas y embriagueces […]” ^16^.

Los hombres morían dentro y fuera de los socavones por muchas causas: explosiones de pólvora, derrumbes en los túneles, desprendimientos de rocas en taludes, silicosis por polvos, cáncer pulmonar, sífilis, cuchilladas en riñas (por mujeres o por oro robado), viruela, tuberculosis, diarrea, dengue grave (conocido como “vómito negro”), *delirium tremens*, quemaduras con fuego, hepatitis causada por el consumo de agua sucia, mordeduras de víboras, rodaduras a precipicios, accidentes con picas o zapas y envenenamiento por comidas descompuestas ^2^. Las condiciones de trabajo en la minería eran siempre penosas y peligrosas, y muy comunes los problemas de espalda por cargar el mineral, además de las enfermedades respiratorias propiciadas por las temperaturas altas ^17^.

Según las descripciones de visitantes de la zona minera, las condiciones de salud pública tampoco eran óptimas en el siglo XIX. En este sentido, en un informe del Boletín de Medicina de la Sociedad Médica de Manizales de principios del siglo XX ^18^, se refiere que las “enfermedades reinantes en nuestros mineros son: paludismo, nefritis, tuberculosis, cirrosis atróficas y anemia” y se insiste en tener mayor rigor científico para su identificación y tratamiento ^18^.

## Enfermedades frecuentes

En 1546 se reportó la primera epidemia de

“[…] pestilencia que abatió la tercera parte de estas grandes provincias y que originaba dolor de cabeza con accidente recio de calentura, pasábase el dolor al oído izquierdo, agravándose de tal suerte, que en dos o tres días pasaban sin remedio de esta vida a los apestados […]” ^6^.

Posteriormente, aparecieron la viruela, el tifo exantemático, la fiebre tifoidea, el sarampión y las paperas. Entre 1702 y 1703, se describe una “epidemia en todos los reales de minas desta jurisdicción a donde se han muerto mucho número de negros” ^19^, precedida por otras dos que originaron una gran mortalidad entre indígenas y esclavos ^19^. En 1792, se reportó en Medellín una epidemia de tabardillo (tifus exantemático) y, entre 1795 y 1796 otra de sarampión, en la cual murieron “muchos niños y gentes mayores” ^20^.

Las enfermedades más comunes en los habitantes del Nuevo Reino de Granada referenciadas por Mutis en 1801 fueron las “dos plagas endémicas” de escrófulas (cotos) y de bubas, seguidas de las “llagas y demás vicios” relacionados con el mal gálico (sífilis)… “ciertamente original del propio clima”. También, era frecuente la lepra “judaica y caratosa”. Por otra parte, en el informe “epidemias anuales” se mencionan la viruela, el sarampión y las disenterías, todas ellas por “desórdenes en el comer, el beber y, en general, todas aquellas debidas a un mal régimen” ^21^.

A principios del siglo XIX. solo existían en la zona los caseríos de Arma y Anserma, casi arruinados, y el Cantón de Supía, que incluía las minas de Marmato. En esta época, los escasos indígenas que quedaban en la zona de la Vega de Supía sufrían los mismos males que durante la Conquista y la Colonia; los accidentes de guerra y de trabajo, las enfermedades infecciosas como la viruela, el tabardillo y la tuberculosis, junto con la picadura de insectos y las mordeduras de serpientes, eran la principal causa de muerte. Se presentaban, además, bubas, sarna y carate, en tanto que la picadura de niguas, garrapatas, chinches y otros insectos, plagas ya endémicas desde el siglo XVI, causaban úlceras ^22^.

Durante el siglo XIX, en Marmato, las enfermedades infecciosas fueron frecuentes. Robledo afirma que, dadas las condiciones en que vivían, los habitantes tenían mayor predisposición a la anemia tropical (por parásitos intestinales) e insiste en que, siendo

“[…] mineros por nacimiento y por necesidad, pues en este municipio apenas se conoce otra manera de ganar el sustento que con el laboreo de las minas, los habitantes viven permanente en inminencia de contagio […]” ^1^.

Asegura también que la tuberculosis, especialmente en su forma de “pleuresía serofibrinosa”, y el paludismo eran endémicos, sobre todo en los trabajadores de las minas. Al igual que Mon Velarde, un siglo y medio antes, insiste en los problemas de piel, especialmente el carate ^1^. Las enfermedades en Marmato entre 1914 y 1915 eran las mismas de la zona minera en el siglo XIX: bronquitis, parásitos intestinales, neumonía, tuberculosis, paludismo y diarrea ^1^.

El carate y la sarna estaban presentes desde la conquista y persistieron, junto con la anemia tropical por uncinariasis, como las enfermedades más frecuentes en la región durante los siglos siguientes. El carate o “la caratea”, denominación que se daba a la “dermatomicosis”, especialmente de la especie llamada “azul” o “asperigulus”, se consideraba la más común entre mineros y se describía como una decoloración parcial o mancha constitucional del cutis que predominaba principalmente en los negros, mulatos y cuarterones ^23^. Los granos, el carate y las gomas constituían el 42 % de las enfermedades de las zonas mineras en el estado soberano del Cauca ^24^.

Bajo el nombre de la enfermedad de las bubas, se referían a diversas entidades que se confundían con la sífilis y con el carate, las cuales eran endémicas en la región, aunque dicha denominación se relaciona más con la frambesia tropical, o pian, otra enfermedad propia de las tierras tropicales húmedas y bajas producida por *Treponema pertenue.* Se considera que fue una enfermedad que las mujeres indígenas contrajeron transmitida por los españoles que acompañaron a Colón ^25^. La disentería, otra enfermedad común en la zona durante el siglo XIX, conllevaba una gran mortalidad infantil, en tanto que la fiebre tifoidea estaba asociada con consumo de agua contaminada, ausencia de saneamiento público, pésimos hábitos higiénicos y malas condiciones ambientales, especialmente el no disponer de sanitarios ^24^.

La tuberculosis se relacionaba con las malas condiciones de la vivienda, las malísimas condiciones higiénicas y el hacinamiento, lo que determinaba que los pobladores “mantienen un aire rumiado” ^1^. Por otra parte, las pleuresías inflamatorias las originaba “el dormir desabrigados, y exponerse estando acalorados a un viento frío y desnudo el pecho” ^26^. Los problemas respiratorios representaban el 7 % de las enfermedades en las zonas mineras del Estado Soberano del Cauca ^24^. Cabe agregar que, por lo regular, la prostitución iba de la mano de la tríada de flagelos sociales endémicos en las zonas mineras: alcoholismo, tuberculosis y sífilis ^1^.

La prostitución es una institución notablemente aceptada en Antioquia y Caldas, puesto que se sabe que cada aldea o población de la región tiene su “barrio de tolerancia”. Se menciona que no se concibe zona minera sin presencia de sífilis o “avería”, como la denominaban los médicos, dados sus efectos a largo plazo ^2^. Entre las enfermedades de transmisión sexual, la sífilis se consideraba una enfermedad hereditaria que producía la degeneración de la “raza” y causaba despoblación, ya que los hombres la transmitían a la esposa y a los hijos, convirtiéndolos en criaturas débiles ^27^. Es evidente que la prostitución fue tolerada en las minas de Marmato; según la historia, las meretrices nunca fueron confinadas oficialmente en “casas de tolerancia”, como sucedía en Medellín. En cuanto a la blenorragia (uretritis gonocócica), se consideraba que afectaba a las mujeres de manera peculiar, pues se infectaban más fácilmente que los hombres y podían permanecer asintomáticas mucho tiempo, especialmente si eran prostitutas ^27^.

El paludismo, denominado “fríos y fiebres”, otra enfermedad endémica en todas las regiones de Colombia ubicadas debajo de los 1.500 msnm fue especialmente virulenta en las hoyas de los grandes valles. En la región bañada por el río Cauca, especialmente en la zona de Maibá (municipio de Supía), era muy común, y se ha reportado que se observaban “formas de carácter pernicioso” ^1^.

La anemia tropical por uncinariasis, también denominada “tun-tun”, anemia verminosa o anemia de mineros, era frecuente en la zona minera de Supía ^28^. Al igual que en la antigua China, también en Marmato la palidez amarillenta o palidez de los mineros de las minas de oro se atribuía al contacto con el metal, y se acompañaba de falta de energía e incapacidad para trabajar ^29^. Se ha estimado que, entonces, el 95 % de la población rural de Colombia andaba descalza y estaba infestada de uncinarias, siendo las zonas interdigitales el sitio de entrada al cuerpo de “candelillas o mazamorras” ^5^.

No hay evidencia de la existencia de lepra en Marmato durante el siglo XIX. El primer caso en la zona se reportó en Salamina, en individuos venidos de Sonsón, en 1841. Una norma publicada el 8 septiembre de 1785 por el oidor Mon y Velarde ordenaba que todos los leprosos de Antioquia fueran llevados a la ciudad de Cartagena ^14^. Sin embargo, la lepra era muy frecuente en el país, especialmente entre negros y mulatos; en un informe de 1883 sobre las impresiones de su viaje al departamento de Antioquia, el Oidor mencionaba la gran frecuencia de “la desastrosa lepra, que las clases bajas creían que se originaba por el exagerado consumo de pescado” ^15^. En el extenso informe del doctor Robledo sobre la lepra en el departamento de Caldas, se señala que el único caso en la región del alto occidente a principios del siglo XX había sido importado de Pácora, de donde el paciente se había trasladado al municipio de Supía ^1^. Pácora se consideró el principal foco de propagación de esta enfermedad durante la segunda mitad del siglo XIX y, a finales de siglo, contaba con un leprosario en las afueras del pueblo ^18^.

La fiebre pútrida, llamada vulgarmente tabardillo (tifus exantemático), se atribuía

“[…] al desaseo, las malas habitaciones, la inacción, e indolencia de semejantes gentes, sus malos alimentos, sus desarreglos, y particularmente por mojarse y permanecer con los vestidos húmedos […]” ^25^.

La epidemia más conocida en el país por sus devastadoras consecuencias fue la de la viruela en 1782, con un grave rebrote en Popayán en 1787 y otro entre 1801 y 1802 ^30^. Después de estos rebrotes, en el cantón de Supía se referencia una epidemia de viruela entre 1840 y 1841, la cual originó la muerte, no solo de la población, sino de soldados que participaban en la llamada Guerra de los Supremos, entre caucanos y antioqueños ^7^. En 1857, hubo otra epidemia de viruela en la región y, aunque la información es poca, es probable que también se presentaran brotes de sarampión, como sucedió en el sur de Antioquia ^28^. Hacia 1896, se construyó un hospital para virolentos en Titiribí en un lapso de solo cuatro días, financiado por la empresa de minas El Zancudo, por la presencia de un minero enfermo procedente de las minas de Marmato ^13^. Debido a la falta de conocimiento y de médicos que las trataran, para enfrentar estas pestes “los habitantes solo contaban con la ayuda de Nuestra Señora de las Mercedes y de San Roque, el santo de las Llagas” ^30^.

Las epidemias de sarampión y paperas en la región de la Vega de Supía y en otras zonas mineras tuvieron diversas presentaciones clínicas, desde “formas simples de tumefacción glandular hasta el caso agudo con altas temperaturas, complicación testicular, deformidad y mal estado general” ^13^.

Para las mordeduras de serpiente se utilizaba la cabeza desecada del animal, la cual se trituraba y se diluía en agua para ser bebida por la persona mordida. Si no se lograba capturar el animal, se les administraba la mezcla de tres yerbas, *Mikania guaco*, bejuco o hierba de serpiente, que fue estudiada por el sabio Mutis en Mariquita durante la Expedición Botánica, la contracapitana y la pareira. Al igual que las parteras, y dada la frecuencia de mordeduras, los esclavos “curanderos de picaduras de víboras” eran tenidos en gran estima por los amos de las minas para la atención de la población del establecimiento minero ^12^. Para tratar las picaduras de gusanos, le sacaban las tripas al animal y se las untaban en la herida, lo que evitaba manifestaciones sistémicas tóxicas ^6^.

Las mutilaciones por accidentes de trabajo eran frecuentes. En un informe notarial de las minas del Cauca a comienzos del siglo XVIII, se hace una relación de 10 % de mancos, 13 % de quebrados, 4 % de cojos y 6 % de bubosos. Las lesiones incluían hernias discales y fracturas, denominadas en la época “quebrado de la rabadilla”, “quebrado del ombligo” y “quebrado de las ingles” ^24^.

Otro problema de la zona eran las tormentas eléctricas y los relámpagos, lo cual se conocía desde tiempo atrás, pues, como decía don Francisco Guillén Chaparro:

“[…] en el propio Cartago; el cielo de aquel pueblo, es que hay muchos serenos y lleve mucho y caen muchos rayos y hai grandes truenos y relámpagos i han muerto gentes […]” ^31^.

Estos fenómenos se consideraban como una causa de muerte en la región, especialmente en las zonas altas de la montaña en los municipios del occidente caldense ^1^. Existe una historia en Marmato sobre un inglés administrador y pagador de un establecimiento minero en 1863, quien patrocinaba orgías con negros; durante una de sus báquicas fiestas, un rayo cayó en un algarrobo que se derrumbó y lo mató ^32^.

Cabría la pregunta de por qué en el alto occidente no había “coto” (bocio), una enfermedad endémica en el país durante el siglo XIX. El coto fue una de las enfermedades más frecuentes durante el siglo XIX en la Nueva Granada, pero no era frecuente en la zona, ya que las salinas abundantes en la región eran todas yodíferas y prevenían su desarrollo; además, fueron reguladas al finalizar el siglo XVIII por el visitador Mon y Velarde, pues eran explotadas al capricho de quien las descubría sin representar ningún ingreso para el fisco ^14^. Posteriormente, Boussingault estudió las causas del coto en las cordilleras de Colombia y explicó su poca frecuencia mediante el análisis químico de las salinas de las minas de la vega de Supía, mencionando que no encontró coto en los indios de tez bronceada ^1^.

## Conclusiones

Las condiciones de salubridad y sus factores determinantes en la población minera de la zona de Marmato, región emblemática de la riqueza del suelo de la Nueva Granada, configuraron una situación de desequilibrio social, cultural, económico y político que impidió el desarrollo armónico de la población nativa. Las dramáticas condiciones de salubridad en la zona predisponían especialmente a enfermedades tropicales infecciosas, frecuentes desde tiempos inmemoriales, las cuales persistieron durante todo el siglo XIX en la región.
